# Wound healing and recurrence in head and neck cancer: a narrative review

**DOI:** 10.37349/etat.2026.1002405

**Published:** 2026-10-15

**Authors:** Micah K. Harris, Wesley L. Cai, Jenny Kim, Vanessa Helou, Joshua D. Smith, Matthew E. Spector

**Affiliations:** General Hospital of Aigio, Greece; Department of Otolaryngology Head & Neck Surgery, University of Pittsburgh Medical Center, Pittsburgh, PA 15213, USA

**Keywords:** wound healing, head and neck cancer, recurrence, surgical site infection, tumor dormancy, immunotherapy, surgical wound fluid

## Abstract

There is a growing body of evidence showing that the postoperative wound healing response shares molecular machinery with tumor pathways. Within larger surgical fields such as breast or colorectal cancer, strong preclinical and clinical evidence exists demonstrating a link between postoperative wound complications and cancer recurrence. Head and neck surgery represents a unique landscape with high rates of surgical site infections (SSIs; 20–30%), driven in part by inherent wound contamination from the aerodigestive tract, in addition to prolonged operative times, malnutrition, smoking, and poor overall health. Despite this, studies addressing postoperative wound healing and head and neck cancer (HNC) recurrence remain fragmented at best, with studies demonstrating significant heterogeneity and conflicting results. A thorough understanding of the oncologic implications of wound complications in HNC is vitally important to improve patient outcomes and identify novel therapeutic strategies. This narrative review seeks to synthesize preclinical and clinical studies linking the postoperative wound environment to HNC recurrence. We also seek to highlight emerging approaches to optimize postoperative wound healing for an oncologic benefit, including the role of surgical drain fluid as a novel bioresource, alternative reconstructive hardware, the role of neoadjuvant immunotherapy, and emerging biomaterials.

## Introduction

In the 19th century, Virchow proposed that cancers arise at sites of chronic inflammation. This concept was formalized more than a century later by Dvorak, who likened tumors to “wounds that do not heal” [1]. Cancer is now widely recognized to co-opt mechanisms of wound repair. It recruits fibroblasts, induces angiogenesis, suppresses immune surveillance, and remodels the extracellular matrix in ways that permit unregulated growth and metastases [1]. While surgery remains indispensable in the management of solid tumors, many of these same pathways are paradoxically reactivated to heal the postoperative wound [2]. Thus, the surgical wound may be argued not to be a biologically neutral space, but rather a dynamic environment that inadvertently provides a favorable (or unfavorable) niche for residual tumor cells.

There is a growing body of in vitro, translational, and clinical evidence establishing a meaningful link between aberrant wound healing and tumor recurrence following oncologic surgery. In breast surgery, postoperative wound healing fluid has been mechanistically shown to stimulate cancer cell proliferation in vitro [3], and large clinical studies have linked poor wound healing with increased locoregional recurrence [4]. Oncologic head and neck surgery represents a unique landscape that warrants special consideration. Unlike other anatomic subsites, head and neck tumors often involve the aerodigestive tract, resulting in direct contamination of the surgical wound with saliva and oral flora during tumor resection. Maintaining separation of the wound from saliva during postoperative healing is a challenge, with rates of salivary fistulas and surgical site infections (SSIs) often exceeding 20–30% [5, 6]. Malnutrition and smoking are also common in this population, further contributing to poor wound healing [5]. Furthermore, the complexity of head and neck tumors necessitates extensive operations with prolonged operative times and microvascular free flap reconstruction [5].

Despite these unique considerations, studies addressing postoperative wound healing and head and neck cancer (HNC) recurrence remain heterogeneous and fragmented. This narrative review seeks to synthesize preclinical and clinical studies linking the postoperative wound environment to HNC recurrence, in addition to highlighting emerging approaches to optimize postoperative wound healing for an oncologic benefit.

## Literature search strategy

A narrative review of the literature was conducted using PubMed, Embase, ClinicalTrials.gov, and Google Scholar. Search terms included combinations of the following: “wound healing,” “cancer recurrence,” “HNC,” and “surgical drain fluid”. Additional terms such as “SSI,” “tumor dormancy,” “field cancerization,” “neoadjuvant immunotherapy,” and “biomaterials” were utilized to capture relevant subtopics. The Boolean search method was applied. Studies that examined the relationship between postoperative wound healing microenvironments and tumor recurrence in HNC were searched, and the search was restricted to the English-language literature. The search focused on studies published within the last 11 years (2015–2026), and the final search was conducted in April 2026; seminal preclinical investigations and landmark clinical studies published prior to this window were included where clinically relevant. Randomized controlled trials, prospective cohort studies, translational in vitro and in vivo studies, and retrospective studies were included. A total of 112 studies were ultimately included. Final study selection was guided by clinical relevance to the review’s scope and was adjudicated by the senior author (MES). This review was not registered and did not adhere to PRISMA or PROSPERO reporting guidelines, consistent with its narrative rather than systematic design.

## Preclinical evidence of wound-mediated recurrence and metastasis

The postoperative wound healing cascade generates a milieu rich in mitogens that are essential for tissue repair but are equally capable of driving tumor cell proliferation and invasion in vitro (Figure 1). Wound healing fluid has been shown to be high in concentrations of mitogens such as transforming growth factor-α, insulin-like growth factor (IGF) I and II, platelet-derived growth factor (PDGF), basic fibroblast growth factor, hepatocyte growth factor, and epidermal growth factor (EGF), all of which play critical roles in cancer development and growth [7].

**Figure 1:**
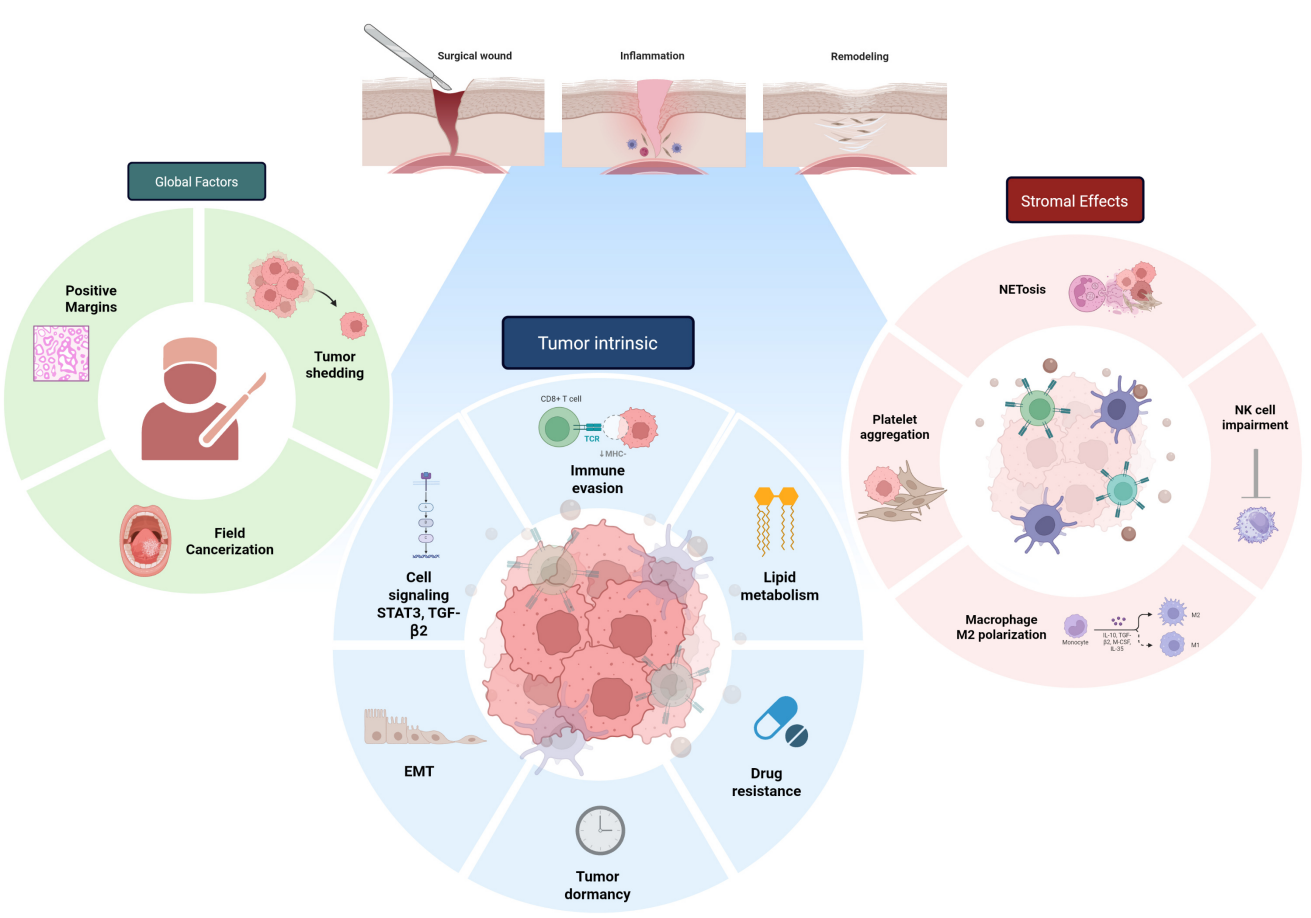
**Cellular factors that may link wound healing and cancer recurrence include global factors (positive margins, field cancerization, tumor shedding), tumor-intrinsic factors (i.e., tumor dormancy, immune invasion), and tumor stromal factors (i.e., NETosis, macrophage polarization, platelet aggregation).** TCR: T cell receptor; MHC-1: major histocompatibility complex-1; STAT3: signal transducer and activator of transcription 3; TGF-β2: transforming growth factor beta 2; EMT: epithelial-mesenchymal transition; NK: natural killer. Created in BioRender. C, W. (2026) https://BioRender.com/mtrv7j2

### Mechanisms of wound-induced proliferation and metastasis

Within the head and neck, Licitra et al. [8] supplemented cell culture medium with postoperative wound fluid collected from 23 patients with HNC (12 who subsequently relapsed, 11 who did not). Every drainage sample tested increased cancer cell proliferation in vitro, and the proliferative effect was significantly greater with fluid from patients who later relapsed. Notably, anti-EGF receptor (EGFR) blockade with cetuximab substantially inhibited this drainage-induced proliferation [8]. Similarly, Scherzad et al. [9] showed that signal transducer and activator of transcription 3 (STAT3) pathway activation mediated increased HNC proliferation in response to wound fluid. These in vitro results are supported by early in vivo evidence. Ekblad et al. [10] found that preincubation of HNC cell lines with postoperative human wound fluid increased tumor uptake in nude mouse xenograft models, effects mediated in part through STAT3 and interleukin-6 (IL-6) signaling. While additional in vivo HNC models are lacking, ample evidence from mouse models demonstrates a link between wound healing and tumorigenicity, further supporting the notion of a pro-tumor wound healing microenvironment [11, 12].

Beyond local proliferative effects, the postoperative wound healing response activates multiple mechanisms that facilitate metastatic spread. Meyer et al. [13] showed that supplementing culture medium with wound fluid upregulated epithelial-mesenchymal transition (EMT) markers, a critical step in the metastatic cascade; moreover, tumor cells incubated with wound fluid showed a significantly higher resistance to cisplatin, potentially reducing the efficacy of systemic therapies. Recently, neutrophil extracellular traps (NETs), which consist of decondensed chromatin and antimicrobial proteins extruded by neutrophils, have been proposed as a novel mechanism for recurrence and metastatic spread [14, 15]. While evolved to sequester pathogens, NETs can also facilitate recurrence by physically trapping and hiding cancer cells [16]. Surgical stress has been shown to encourage platelet-tumor cell aggregate entrapment within NETs via Toll-like receptor 4-extracellular signal-regulated kinase 5-glycoprotein IIb/IIIa (TLR4-ERK5-GPIIb/IIIa) signaling, thereby protecting circulating tumor cells from immune clearance [16]. Surgery-induced NETs are also capable of driving distant metastatic seeding in murine models through various mechanisms such as reprogramming of lipid metabolism, with early correlative studies in humans beginning to emerge [15, 17].

### Surgery-induced immune suppression

Surgery is recognized to induce a state of systemic and local immunosuppression, which may allow residual cancer cells to evade immune surveillance in the perioperative period [18]. Natural killer (NK) cell dysfunction is a well-characterized mediator of this phenomenon. In murine models, surgical stress impairs NK cytotoxicity through various mechanisms including prostaglandin-mediated and catecholamine-mediated pathways [19]. A possible coagulation-dependent mechanism for this impaired cytotoxicity was elucidated by Seth et al. [20], who demonstrated in murine models that surgery induced fibrin and platelet clot formation around circulating tumor cell emboli, physically shielding them from NK cell-mediated detection and destruction; interestingly, perioperative anticoagulation reversed much of this protective effect. Surgical immune suppression may also drive metastatic spread, as adoptive transfer of surgically stressed NK cells into NK-deficient murine hosts has been found to increase pulmonary metastases [21].

Surgery also triggers expansion of myeloid-derived suppressor cells (MDSCs) and suppression of cytotoxic T cells. Ananth et al. [22] showed that surgical stress abrogated pre-existing T cell-mediated anti-tumor immunity in a mouse model of cancer vaccination, with reduced CD8+ T cell cytokine production and concurrent expansion of pro-tumorigenic MDSCs. More recently, Angka et al. [23] used single-cell RNA sequencing to demonstrate that surgery-induced MDSCs upregulated phosphoinositide 3-kinase gamma (PI3K-γ) signaling, and that PI3K-γ inhibition reduced postoperative metastases in murine models, identifying a potentially actionable immunologic target in the perioperative period.

### Radiation-induced immune changes

Many HNC patients receive radiation, whether definitively, as induction or neoadjuvant treatment, or in the adjuvant setting. Radiation is known to impair wound healing, which by extension may have oncologic implications [24]. Radiation triggers release of transforming growth factor beta 2 (TGF-β2), differentiation of fibroblasts into myofibroblasts, deposition of collagen-rich matrix, and reduction of matrix metalloproteinase (MMP) activity [25]. This results in a hypoxic, hypovascular, and hypocellular tissue state that impairs wound healing and leaves the area susceptible to infection. These changes can persist years after completion of radiation therapy, with Bian et al. showing that irradiated dermal fibroblasts retain “radiation memory” in the form of epigenetic reprogramming that impairs their motility and wound-repair capacity long after exposure [26]. In fact, many of the same inflammatory signals seen post-radiation are the same as those implicated in tumor recurrence. TGF-β2, often cited as the main regulator of radiation fibrosis, is also implicated in HNC dormancy and dissemination [27]. This raises the underexplored possibility that the fibrotic, hypoxic, chronically inflamed post-radiation wound bed may represent a distinctly permissive niche for residual disease. Despite this, the interaction between radiation-induced wound pathology and HNC recurrence remains essentially unstudied, and no study to date has stratified the wound-complication-recurrence relationship by prior radiation exposure. Given that radiation is nearly ubiquitous in HNC management, dedicated investigation of how the irradiated wound microenvironment influences both healing and oncologic outcomes represents a critical and clinically actionable gap.

## Etiology of residual disease

Preclinical evidence has convincingly shown that the wound healing microenvironment has the capacity to be a favorable environment for cancer cell growth. Current proposed mechanisms require viable malignant or pre-malignant cells to be present in the wound, however. Here, we review etiologies of residual disease and pre-malignant cells that may be subject to stimulation by the wound environment.

### Microscopic residual disease and tumor dormancy

Despite high rates of negative margins on pathology (> 90%), recurrence of HNC occurs in up to 30% of patients, with locoregional recurrence predominating, suggesting that microscopic disease remains in the wound bed [28]. Various overlapping mechanisms have been proposed. One prevailing etiology is that pathologic examination may fail to identify residual disease. In a landmark study by Brennan et al. [29] in 1995, molecular markers were used to show that 53% of specimens with histologically negative margins contained clonal populations of tumor cells. More recent studies have reported false negative rates up to 38% [30]. Moreover, positive margins remain a challenge in the head and neck, with rates of up to 15–30% being reported [31–33].

Another common theory is tumor dormancy, in which residual cancer cells persist in a quiescent, non-proliferating state. Dormant cells survive surgical stress and evade immune detection, retaining the capacity to re-enter the cell cycle depending on environmental signals [34]. The postoperative wound is rich in inflammatory cytokines, which may serve as a trigger for tumor dormancy, reactivation of dormant cells, or proliferation of residual viable cells [12]. In HNC, the cytokine TGF-β2 has been shown to induce an atypical EMT state that promotes tumor dormancy and immune cell evasion via mitogen-activated protein kinase (MAPK)-dependent signaling [27]. Conversely, common wound healing factors such as M2-polarized macrophages and pro-inflammatory cytokines (i.e., IL-8) can reawaken dormant cells through MAPK and ERK signaling [35]. These data suggest that the wound healing microenvironment plays a dynamic role in manipulating dormancy of residual cancer cells, ultimately contributing to late recurrence.

### Intraoperative tumor shedding

Beyond leaving microscopic residual disease or dormant tumor cells, surgical manipulation itself liberates tumor cells into the wound bed, draining lymphatics, and systemic circulation. Early work by Hansen et al. [36] showed that in 61 patients undergoing oncologic surgery for multiple tumor types including HNC, blood recovered from the surgical field contained malignant cells in 57 cases; importantly, these cells retained ex vivo tumorigenicity in a xenograft model, supporting their biological viability. More recently, Mastronicola et al. [37] detected circulating tumor cells in surgical drain fluid on postoperative day 1 in 70% of patients undergoing head and neck surgery, demonstrating direct seeding of the wound environment by viable tumor cells. Shed tumor cells are not limited only to the local wound, but are also found in the peripheral circulation. Jatana et al. [38] demonstrated a greater than 6-fold increase in circulating tumor cells in the peripheral blood of HNC patients following tumor resection, with 8% of patients who were circulating tumor cell-negative preoperatively converting to positive after surgery.

Despite this evidence, establishing a direct causal link between disseminated cells deposited in the wound and subsequent development into clinically evident recurrence remains inherently difficult. Correlative clinical evidence, however, supports this concept. Stomal recurrence following total laryngectomy is more frequent when tracheostomy is performed prior to resection, implicating direct implantation of tumor cells [39, 40]. Similarly, percutaneous endoscopic gastrostomy (PEG) site metastases occur in approximately 0.5% of patients with upper aerodigestive tract malignancies, with the “pull through” technique, which drags the tube through the tumor-bearing upper aerodigestive tract, increasing the risk of PEG stoma recurrence [41, 42].

### Field cancerization

Another mechanism by which the postoperative wound environment may promote disease relates to field cancerization. Originally described by Slaughter et al. [43], field cancerization posits that carcinogen exposure (i.e., smoking) induces widespread molecular alterations across a region, predisposing to multiple independent primary malignancies. This phenomenon is well-established in HNC, where rates of second primary tumors approach 10–15% over 10 years [44]. Preneoplastic fields can extend > 7cm from the primary tumor [45], with Tabor et al. [46, 47] detecting genetically altered fields beyond the surgical resection margins in 36% of HNC patients; in 61% of cases with locoregional recurrence, the recurrent tumor originated from a residual precursor field rather than the primary tumor site. Field cancerization raises the concern that postoperative wound healing factors may not only act on residual tumor cells but also on dysplastic precancerous mucosa. Murine wounding models have demonstrated that neutrophils recruited to the wound are capable of redirecting proliferating signaling toward pre-neoplastic cells via prostaglandin E2-mediated signaling, accelerating malignant transformation [11]. Modulating the postoperative wound healing environment may therefore represent a rational therapeutic strategy to limit not only the growth of residual tumor cells, but also malignant transformation of premalignant cells within the surrounding dysplastic epithelium.

## Clinical evidence linking wound complications and recurrence

Despite strong preclinical evidence demonstrating a link between the postoperative wound and tumor recurrence, translating these findings into the clinical setting has been met with mixed results. The most mature clinical evidence examining wound complications and recurrence in the clinical setting comes from breast surgery. In a cohort of 1,065 mastectomy patients, Murthy et al. [4] demonstrated a significant association between postoperative wound complications (9% of patients) and systemic recurrence [hazard ratio (HR) 2.87], an effect that persisted after controlling for tumor stage and risk factors. More recently, a systematic review of 10 retrospective cohort studies encompassing 37,657 mastectomy patients identified a relationship between wound complications and worse 5-year recurrence-free survival (RFS; HR 1.48) [48]. However, 5 of these studies failed to demonstrate a significant relationship, and there was a high degree of heterogeneity amongst the studies (*I^2^* = 95%) [48]. This heterogeneity carries into HNC-specific studies (Table 1) [49–61].

**Table 1:** Review of published literature investigating the relationship of SSI on HNC survival outcomes.

**Author, year**	***n***	**% of free flaps in cohort**	**Primary site**	**Complication**	**Findings**	**Study design**	**Level of evidence**	**Use of a standardized SSI definition**
Schantz et al. 1980 [49]	201	Not reported	Larynx	SSI	Improved 5-year OS (44% vs 31%, *P*-value not reported).	Retrospective	III	No
Jackson and Rice 1990 [50]	100	Not reported	All HNC sites	SSI	No association with RFS (43% vs 64%, *P* = 0.20).	Retrospective	III	No
Grandis et al. 1992 [51]	134	Not reported	All HNC sites	SSI	Worse DFS (40% vs. 55%, *P* = 0.04)	Retrospective	III	No
Rodrigo and Suárez 1998 [52]	158	Not reported	Larynx	SSI	Increased recurrence (57% vs 27%, *P* = 0.04); no association with 3-year OS (57% vs. 73%, *P* = 0.20).	Retrospective	III	No
Penel et al. 2004 [53]	95	33 (34.7%)	HNC violating the aerodigestive tract	SSI	No association with local recurrence (25.5% vs. 22.9%, *P* = 0.8) or death (53.2% vs. 56.3%, *P* = 0.39)	Prospective	II	No
González-Márquez et al. 2012 [54]	129	Not reported	Larynx and hypopharynx	SSI	Worse 3-year DSS (HR 1.9, 95% CI 1.01–3.60, *P* = 0.04).	Retrospective	III	No
Hirakawa et al. 2013 [55]	277	154 (55.6%)	All HNC sites	SSI	No association with 5-year OS (46.7% vs 54.2% SSI, *P* = 0.57)	Retrospective	III	No
Mascarella et al. 2019 [56]	114	Not reported	Oral cavity	Postoperative infection, including systemic	Worse 5-year OS (24.1% vs. 65.2%, *P* < 0.0001)	Retrospective	III	No
de Almeida et al. 2019 [57]	551	Not reported	Oral cavity	SSI	No association with 5-year RFS (51% vs. 62%, *P* = 0.21) or OS (63% vs. 67%, *P* = 0.71)	Retrospective	III	No
Davies et al. 2021 [58]	550	135 (24.5%)	Larynx (salvage laryngectomy)	Pharyngocutaneous fistula	Associated with distant metastases (HR 2, 95% CI 1.22–3.27, *P* = 0.005).	Multicenter retrospective	III	No
Hiraoka et al. 2022 [59]	67	67 (100%)	Oral cavity	SSI	Worse OS (HR 2.69, 95% CI 1.038–6.970, *P* = 0.04)	Retrospective	III	No
Thiagarajan et al. 2023 [60]	245	83 (33.8%)	All HNC sites	SSI	Worse DFS (OR 3.75, 95% CI 1.799–7.849, *P* < 0.001)	Retrospective	III	No
Yonaga et al. 2026 [61]	346	346 (100%)	All HNC sites	Clavien-Dindo grade ≥ IIIa SSI	Worse OS (HR 3.53, CI 95% 1.8-7.01, *P* < 0.001) and RFS (HR 2.1, 95% CI 1.2–4.1, *P* < 0.001)	Retrospective	III	Clavien-Dindo

DFS: disease-free survival; HNC: head and neck cancer; RFS: recurrence-free survival; SSI: surgical site infection; OR: odds ratio; OS: overall survival.

The earliest study by Schantz et al. in 1980 [49] paradoxically reported improved survival with postoperative wound infection. A proposed mechanism for such findings is the “Coley’s toxin” phenomenon, whereby bacterial pathogen-associated molecular patterns prime and activate the adaptive immune system that may then target cancer cells [49]. However, these data have been outweighed in most modern cohorts. Subsequent work by Grandis et al. [51] found that wound infection independently predicted decreased survival with an odds ratio (OR) of 2.4, while more recent work by González-Márquez et al. [54] and Mascarella et al. [56] confirmed wound infection as an independent prognostic factor for disease-specific survival (HR 2.54). Similarly, Yonaga et al. [61] demonstrated that Clavien-Dindo grade > IIIa SSI independently predicted both worse overall survival (OS; HR 3.539) and RFS (HR 2.095) in a group of 346 free flap patients. In the largest study of oral cavity cancer patients (*n* = 551), however, de Almeida et al. [57] found that while patients who developed postoperative wound infection (*n* = 98) had a higher risk of recurrence and death on univariate analysis, these relationships did not persist when controlling for tumor stage and pathological risk factors on multivariate analysis, though there was an increased trend (*P* = 0.10) toward distant metastases. Similarly, Davies et al. [58] conducted a large multi-institutional review of 550 total laryngectomy patients, finding that patients who developed pharyngocutaneous fistula (*n* = 127) had a 2-fold higher rate of distant metastases, though the rates of disease-free and OS were similar. In these studies, however, wound complications lacked standardized definitions, likely contributing to the observed heterogeneity.

Postoperative wound complications can also delay the initiation of adjuvant therapy, which can indirectly impair oncologic outcomes. The National Comprehensive Cancer Network guidelines recommend that postoperative radiation begin within 6 weeks of surgery [62]. However, about 55% of patients fail to meet this benchmark [63]. Janz et al. [64] demonstrated that patients with wound complications were nearly 3 times more likely to experience delayed postoperative radiation (OR 2.79). Similarly, Thiagarajan et al. [60] identified SSI as the single strongest predictor of delayed adjuvant treatment initiation (OR 3.79) in a cohort of 245 patients with upper aerodigestive tract squamous cell carcinoma. The oncologic consequences of these delays are well established. In a landmark National Cancer Database analysis of 41,291 HNC patients, Graboyes et al. [63] demonstrated that initiating postoperative radiotherapy beyond 6 weeks was independently associated with decreased OS (adjusted HR 1.13).

There are several possible explanations for the heterogeneity seen in these clinical studies. A major confounder is the complexity of wound healing, which can be influenced by numerous factors, including preoperative radiation, hypothyroidism, diabetes, nutritional status, perioperative transfusion, and tumor characteristics, which are often difficult to control for [65–67]. Survival outcomes are equally complex and are influenced by surgical technique and adjuvant therapies. Thus, linking the two variables becomes inherently complex. Human papillomavirus-positive and negative tumors have distinctly different biologies, immunologic backgrounds, treatment, and prognosis [68]. However, no study to date has compared the wound-recurrence relationship between the groups.

Free flap reconstruction is also an important metric to report. Receiving a free flap indicates the presence of a large surgical wound that is at higher risk of healing complications. Conversely, free flaps can improve wound healing through healthy vascular support, delivery of growth factors, and immune cell support. The immunologic signature of flap transfer differs from non-reconstructed surgical beds—analyses of postoperative wound exudate from free flap reconstructed breast defects demonstrated a blunting of the proinflammatory cytokine surge and a shift toward an anti-inflammatory profile, though the same has not been explored in HNC [69]. This is supported clinically by lower rates of salivary fistula and wound-healing complications observed with free flap utilization, as shown by Hanasono et al. in oral cavity resections and corroborated by Moreno et al. for laryngectomy defects [70, 71]. However, it should be noted that whether these differences contribute to improved oncologic outcomes remains an unanswered mechanistic question. Only 6 of the 13 published studies on wound healing and HNC recurrence reported the number of free flaps, with only 2 studies including a cohort of free flap patients alone (Table 1).

Finally, diagnosing wound complications relies on clinical acumen, which can often be subjective and imprecise. Only one of the 13 studies examining wound healing and recurrence in HNC used an established classification (Clavien-Dindo) scale (Table 1). To facilitate better comparisons, the use of standardized classifications should be included in future studies. Wound complication scores such as InCISE have been proposed but have yet to be integrated into clinical practice [72].

## Clinical interventions and future directions

Despite heterogeneous clinical data, there is a compelling biologic rationale supporting a link between postoperative wound healing and recurrence in HNC. Translating this knowledge into clinical intervention requires strategies that reduce wound complications but do so with an explicit awareness of oncologic consequences. Here, we review emerging and investigational approaches to wound healing optimization in head and neck surgery, with particular attention to their potential oncologic implications.

### Surgical wound fluid

Surgical wound fluid is an exciting bioresource that allows direct sampling of the postoperative wound healing environment (Figure 2). Surgical drains are routinely placed in the neck after major head and neck surgery to drain wound effluent over subsequent days. Because wound fluid accumulated in the bulb of the surgical drain is otherwise discarded, use of drain fluid does not require additional interventions or modification of existing clinical infrastructure. Traditional blood-based labs such as complete blood count are inherently nonspecific, reflecting systemic inflammation rather than localizing to the surgical wound itself. As such, peripheral blood markers cannot differentiate between SSIs and other causes of postoperative inflammation (e.g., pneumonia, urinary tract infections, or blood stream infections). Unlike blood samples, which often dilute molecular signals, surgical wound fluid provides a liquid biopsy that is measured directly from the surgical bed, offering improved specificity compared to peripheral blood markers [73]. Drain fluid-based markers of inflammation or measurement of cell types within the wound may ultimately prove superior to blood-based labs to monitor SSI, though additional work is needed.

**Figure 2:**
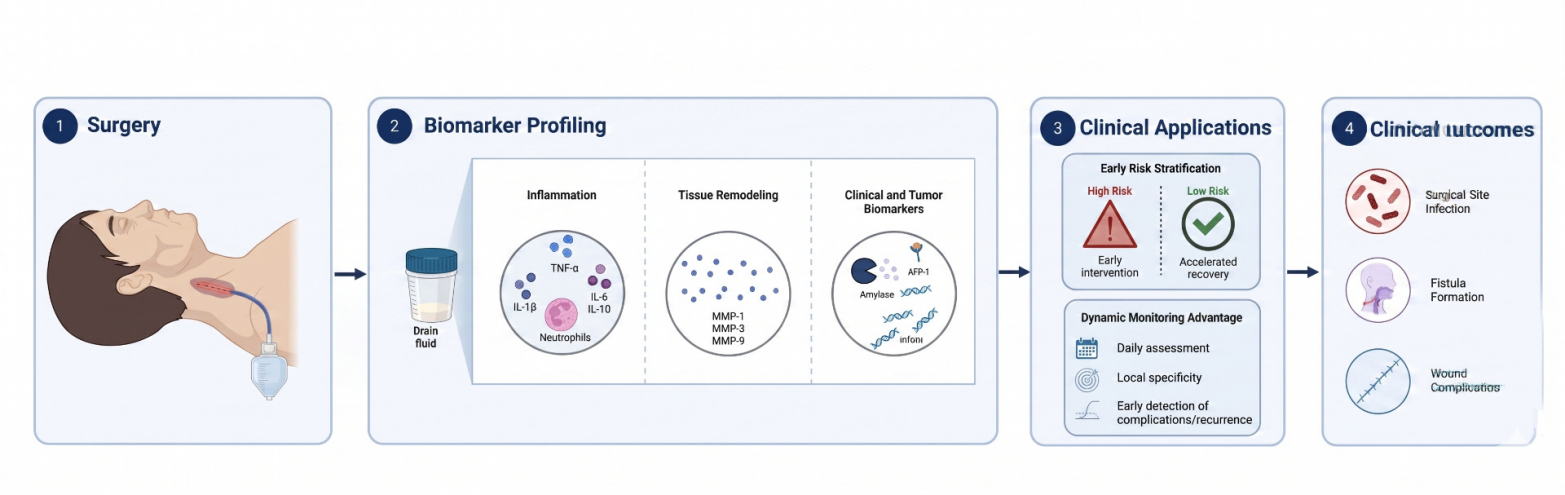
**Surgical wound fluid as a localized bioresource of the postoperative wound healing environment.** TNF-α: tumor-necrosis factor alpha; IL-6: interleukin-6; MMP-1: matrix metalloproteinase-1. Created in BioRender. Helou, V. (2026) https://BioRender.com/puwiizd

Despite the clinical relevance and potential, limited attention has been paid to wound fluid-derived markers of wound complications. The majority of data comes from retrospective, level II/III clinical studies. Amylase, an enzyme found in human saliva, is the most heavily investigated. Drain fluid amylase was first reported to correlate with pharyngocutaneous and orocutaneous fistula in 1984 [74]. More recent data have shown drain fluid amylase to be a particularly useful marker to identify patients at low risk of fistula, with negative predictive values of > 90% [6, 75]. Others have examined more traditional wound healing factors. Lassig et al. [76] measured a panel of wound healing factors in the surgical drains of HNC patients 24 hours after surgery, showing IL-1β, IL-8, and MMP-9 to be higher in the wound fluid of those who went on to have wound complications, though predictive strengths were not reported. Additional studies have shown postoperative day 1 TNF-α to predict SSI following neck dissection in HNC patients [77].

Despite these promising data, drain fluid-derived markers of wound complications have not yet been integrated into clinical investigations. A potential avenue is to utilize drain fluid biomarkers to stratify patients into low- and high-risk groups early in the postoperative course, possibly allowing for early intervention in patients at a high-risk of developing wound complications, whereas those at a low-risk may be placed on an accelerated recovery protocol. Moreover, surgical drain fluid may continue to be utilized as a tool to study the postoperative wound healing microenvironment, providing further insights into the relation of wound healing and cancer recurrence.

Beyond markers of wound complications, surgical drain fluid has been utilized as a liquid biopsy resource to identify predictors of recurrence, further underlining the relationship between the postoperative wound environment and recurrence. Lassig et al. [78] showed that MMP-1, MMP-3, and soluble fms-like tyrosine kinase-1 levels in the wound fluid were associated with recurrence in a small cohort of 20 patients. The most convincing data was produced by Lazare et. al., who utilized a tumor-based assay to demonstrate that cell-free tumor DNA mutations in surgical drain fluid strongly predicted tumor recurrence, outperforming and complementing traditional pathologic risk criteria [73, 79]. Given these promising findings, surgical drain fluid may prove to be a powerful bioresource to investigate both wound complications and recurrence.

### Mandible hardware

Mandible hardware represents another understudied area within head and neck surgery that may lend itself to improving wound healing complications. Segmental mandibulectomy is often required after resection of advanced malignancies, and reconstruction plates are typically used for reconstruction, with or without vascularized bone flaps [80]. Though titanium plates are considered standard of care, clinical studies have shown hardware-related complications to occur in anywhere from 30–50% of patients [81, 82]. These complications, such as plate exposure, infection, plate fracture, and screw loosening, can result in chronic, non-healing wounds that are often contaminated by saliva due to intraoral communication. Moreover, preclinical studies have shown that the high atomic number of titanium causes backscatter of radiation, resulting in significant dose enhancement at the hardware-tissue interface, while shadowing reduces the radiation dose underneath the plate [83–85]. Despite these risks, no studies have yet examined a link between plate complications and cancer recurrence or delay to adjuvant therapy initiation.

Alternative plating material may be a promising method of reducing plate-related wound complications and simultaneously improving oncologic control. Biodegradable magnesium plates are being tested in the preclinical setting and have demonstrated similar biomechanical performance to titanium, though clinical testing is pending [86]. Perhaps the most promising material is carbon-reinforced polyetheretherketone (CFR-PEEK). In the neurosurgery field, CFR-PEEK is routinely used in cranioplasty and spinal fusion [87]. CFR-PEEK offers several advantages, including radiolucency, biocompatibility, and minimal radiation dose perturbation [88, 89]. Clinical experience with CFR-PEEK plates in head and neck reconstruction has shown potential in preclinical models, though clinical applications remain limited to case series [90, 91]. More rigorous biomechanical testing and structured clinical evaluations using alternative plating materials should be pursued in order to decrease wound complications and improve radiation dosimetry in mandibulectomy patients.

### Neoadjuvant and adjuvant immunotherapy

Immunotherapy has fundamentally reshaped the field of HNC. In 2016, the anti-programmed cell death protein-1 (anti-PD-1) monoclonal antibodies pembrolizumab and nivolumab were approved as first-line therapy for recurrent/metastatic head and neck squamous cell carcinoma [92]. In June 2025, the FDA approved neoadjuvant pembrolizumab for resectable locally advanced HNC following the results from the phase III KEYNOTE-689 trial demonstrating an 11% improved event-free survival with neoadjuvant immune checkpoint inhibition [93, 94]. However, immune checkpoint inhibitors can interfere with wound healing through disruption of programmed death-ligand 1 (PD-L1) mediated inflammation resolution, impaired polarization from pro-inflammatory M1 to reparative M2 macrophages, disrupted fibroblast function, and T cell-mediated tissue injury [95].

While the majority of clinical data are reassuring, findings have been heterogeneous. In particular, patients undergoing complex surgeries may be at higher risk of postoperative wound complications. A multi-institutional propensity-matched study by Philips et al. [96] (*n* = 462) found no difference in postoperative complications in those who received neoadjuvant immune checkpoint inhibition, though only 28.9% underwent free flap reconstruction. A large retrospective cohort of 692 patients by Chen et al. [97] interestingly found that major wound complications were lower in the neoadjuvant immunochemotherapy group (9.2% vs. 17.8%), possibly reflecting tumor downstaging, though again a minority (36%) required free flap reconstruction. In the only study that included solely free flap reconstruction patients (*n* = 132), Mays et al. [98] showed that those who received neoadjuvant immunotherapy (62%) demonstrated higher rates of major wound complications (OR 3.7), suggesting that vigilance is warranted for patients undergoing more complex surgeries.

Ongoing trials are investigating neoadjuvant dual immune checkpoint blockade, such as anti-PD-1 in conjunction with anti-cytotoxic T-lymphocyte-associated protein 4 (anti-CTLA4) or anti-lymphocyte activation gene-3 (anti-LAG-3) [99]. Early data has shown higher rates of immune-related adverse effects with dual inhibition, though postoperative wound healing complications have not yet been investigated. As new immunotherapy agents emerge and indications for neoadjuvant therapy continue to evolve, the impact on postoperative wound healing should be closely evaluated.

Moreover, emerging evidence suggests that the extent of neck dissection may influence the efficacy of immunotherapy. Saddawi-Konefka et al. [100, 101] demonstrated in murine models of HNC that ablation of tumor-draining lymph nodes eliminated the tumor response to immune checkpoint inhibition and worsened OS. These results indicate that lymph nodes are not merely metastatic waystations but are hubs for immunotherapy response, supporting lymphatic-preserving approaches. As such, some surgeons in the field are anecdotally moving toward limited neck dissections, in which less extensive neck dissections are performed to theoretically preserve systemic antitumor immunity. This “supraselective neck dissection” approach may lend itself to less extensive surgical wounds and perhaps accelerated postoperative wound healing, though currently there is a lack of clinical data to support such practice. As this aspect of the field continues to evolve, the effect of neck dissection extent on the postoperative wound should be examined.

### Emerging wound healing interventions and oncologic implications

Following established perioperative measures is fundamental to any strategy aimed at reducing wound complications. Meticulous surgical technique, watertight wound closure, preoperative nutrition, appropriate antibiotic prophylaxis, and adherence to Enhanced Recovery After Surgery (ERAS) protocols are just a few interventions that form the foundation of wound healing optimization [5]. Beyond these standards, there is a growing body of evidence describing novel interventions to improve wound healing that warrant consideration.

Intraoperative perfusion assessment is a promising avenue to reduce wound complications. The SPY perfusion assessment system (LifeCell Corp., Branchburg, NJ, USA) uses indocyanine green angiography to provide real-time intraoperative visualization of tissue perfusion, enabling surgeons to identify and resect poorly perfused zones prior to wound closure. Clinical data from breast surgery suggest SPY-guided resection reduces skin flap necrosis and wound complication rates [102]. Within head and neck surgery, adequate tissue perfusion is critical to prevent salivary fistulas, which often develop despite meticulous pharyngeal closure and free flap reconstruction. Early clinical data have shown that patients who develop salivary fistula demonstrate lower perfusion of the native pharyngeal mucosa intraoperatively, though prospective trials are needed to validate these findings [103].

Novel biomaterials represent perhaps the most rapidly evolving frontier in wound healing science, with potential applications in head and neck surgery that extend beyond simple tissue coverage, though it should be noted that these remain in the preclinical stage. Fish skin-derived acellular matrices, ex vivo-produced oral mucosal equivalents, bioactive glasses, Poly(lactic-co-glycolic acid) (PLGA)-based drug delivery scaffolds, and electrospun membranes have each demonstrated capacity to modulate the local inflammatory milieu and promote organized mucosal regeneration [104]. Injectable reactive oxygen species-scavenging hydrogels represent a particularly intriguing approach: by neutralizing the oxidative stress that perpetuates chronic wound inflammation, these materials may shift the wound microenvironment away from a pro-tumorigenic state toward one more conducive to orderly healing [105]. Hydrogels that mimic the extracellular matrix architecture further support local tissue regeneration by providing a scaffold for fibroblast and keratinocyte ingrowth [106]. Bioprinting technology, which enables spatially precise deposition of cells and bioactive molecules, offers the future possibility of constructing patient-specific wound-healing constructs tailored to the complex three-dimensional defects generated by ablative head and neck surgery [107].

Autologous platelet concentrates (APCs) are regenerative products prepared by centrifugation of a patient’s own blood. Re-infusion releases concentrated growth factors [PDGF, TGF-β2, vascular endothelial growth factor (VEGF), EGF, and IGF] that promote angiogenesis, fibroblast recruitment, and re-epithelialization. APCs can be divided into four groups by leukocyte content and fibrin architecture—pure platelet-rich plasma (P-PRP), leukocyte- and platelet-rich plasma (L-PRP), pure platelet-rich fibrin (P-PRF), and leukocyte- and platelet-rich fibrin (L-PRF) [108, 109]. Within head and neck surgeries, a randomized controlled trial of 67 total laryngectomy patients found that PRF applied to the pharyngeal repair significantly reduced pharyngocutaneous fistula (5.7% vs. 31.3%), a randomized trial in partial parotidectomy reported reduced salivary fistula with perioperative PRP, and a 2026 systematic review across otologic, rhinologic, and laryngologic surgery found consistent improvements in tissue healing attributed to downregulation of pro-inflammatory cytokines and MMP-9 modulation [110–112]. It should be considered that in theory, the same growth factors in APCs may stimulate residual tumor growth as described earlier, though no studies have investigated this aspect.

Many other wound healing interventions are on the horizon. As new interventions and biologics are tested clinically, monitoring the oncologic impact of improved wound healing will be critical.

## Limitations

Several limitations should be considered when interpreting the findings of this review. First, as a narrative review, the methodology did not strictly adhere to PRISMA or PROSPERO reporting guidelines typically utilized for systematic reviews or meta-analyses. The inclusion of the 99 studies was guided by clinical relevance and adjudicated by the senior author, which introduces an inherent degree of subjectivity and potential selection bias. Consequently, the search strategy was not exhaustive, and the review does not cover every available literary source on the topic. Furthermore, this manuscript serves to synthesize existing preclinical and clinical evidence rather than presenting findings from an original prospective study conducted by the authors. Finally, the overarching conclusions of this review are constrained by the quality of the underlying literature itself, which is marked by significant heterogeneity, conflicting results, a heavy reliance on retrospective cohorts, and a notable lack of standardized definitions for evaluating SSIs.

## Conclusion

The relationship between postoperative wound healing and HNC recurrence is supported by a compelling body of preclinical evidence showing that the surgical wound contains healing factors capable of stimulating tumor cell growth and dormancy reactivation. Clinical evidence linking wound complications to recurrence remains heterogeneous, likely hindered by inconsistent definitions, subjective diagnoses, and an inherent difficulty discerning wound-related effects from underlying tumor biology. Nonetheless, several promising strategies exist to modulate the postoperative wound with potential oncologic benefit, including surgical wound fluid biomarker profiling, alternative mandible plate materials, and novel biomaterials. As oncologic therapies for HNC continue to evolve, continued characterization of wound outcomes will be essential to not only optimize functional recovery, but to improve disease control and survival outcomes.

## Abbreviations

anti-PD-1: anti-programmed cell death protein-1
APCs: autologous platelet concentrates
CFR-PEEK: carbon-reinforced polyetheretherketone
DFS: disease-free survival
EGF: epidermal growth factor
EMT: epithelial-mesenchymal transition
HNC: head and neck cancer
HR: hazard ratio
IGF: insulin-like growth factor
IL-6: interleukin-6
MDSC: myeloid-derived suppressor cell
MMP: matrix metalloproteinase
NETs: neutrophil extracellular traps
NK: natural killer
OR: odds ratio
OS: overall survival
PDGF: platelet-derived growth factor
PEG: percutaneous endoscopic gastrostomy
PI3K-γ: phosphoinositide 3-kinase gamma
P-PRF: pure platelet-rich fibrin
P-PRP: pure platelet-rich plasma
RFS: recurrence-free survival
SSIs: surgical site infections
STAT3: signal transducer and activator of transcription 3
TGF-β2: transforming growth factor beta 2

## Declarations

### Copyright

© The Author(s) 2026.
